# Congenital Retinal Macrovessel Without Any Ophthalmic Complications: A Case Report

**DOI:** 10.7759/cureus.44500

**Published:** 2023-08-31

**Authors:** Vijaya Natarajan, Sonia Mathew, Adhithya N Balaji, Jayshree Ahirrao-Mutta

**Affiliations:** 1 Ophthalmology, NMC Specialty Hospital, Abu Dhabi, ARE; 2 Oncology, Università Cattolica del Sacro Cuore, Rome, ITA; 3 Ophthalmology, NMC Royal Family Medical Centre, Abu Dhabi, ARE

**Keywords:** foveal avascular zone, spectral domain optical coherence tomography, optical coherence tomography angiography, aberrant retinal vessel, congenital retinal macrovessel

## Abstract

Congenital retinal macrovessels (CRMs) are a rare entity. They are usually unilateral, abnormally large, and aberrant vessels. Although the majority of the patients are asymptomatic, CRMs may affect vision if they are associated with pigmentary changes at the macula, foveolar cysts, central serous retinopathy, macular hemorrhage, or if the macrovessel crosses the fovea.

Here, we present the case of a young female who came for a routine ophthalmological evaluation. She was asymptomatic, and the macrovessel was an incidental finding. Visual acuity and slit lamp examination were normal, and dilated fundus evaluation was normal except for CRM in the right eye. Optical coherence tomography angiography imaging helped visualize the depth of the vessel and the analysis of the architecture of the foveal avascular zone.

## Introduction

Congenital retinal macrovessels (CRMs), first reported and described in 1869, are aberrant vessels, typically veins larger than the usual size, crossing the horizontal raphe in the macular region [1]. Although CRM is considered a benign condition, its clinical significance lies in its association with visual disturbances and complications such as macular edema, retinal vein occlusion, and hemorrhages. While the exact etiological mechanisms leading to CRM formation remain unclear, there is evidence suggesting a multifactorial origin involving genetic predisposition and embryonic vascular development anomalies. CRM now can be examined by optical coherence tomography angiography (OCTA), which allows for non-invasive imaging of the retinal vasculature [1].

## Case presentation

**Figure 1:**
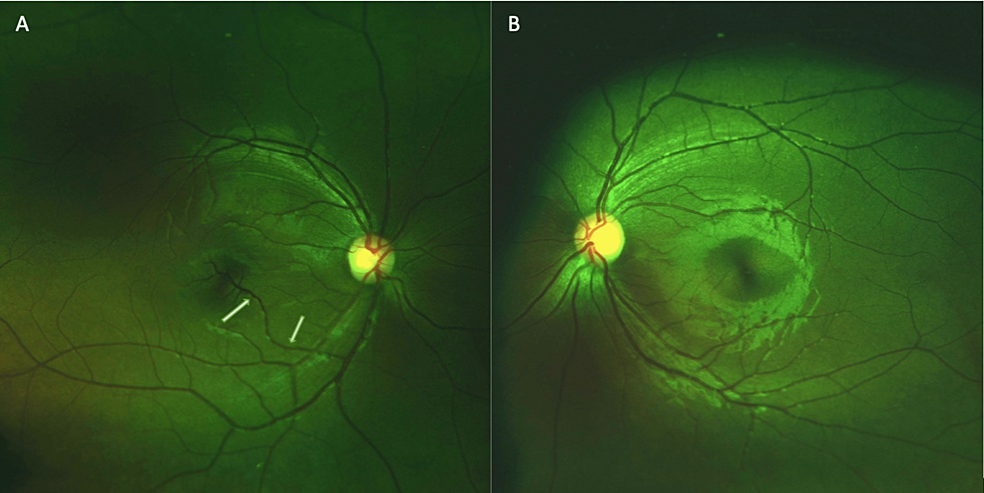
Color fundus photograph of the right (A) and left (B) eyes. (A) White arrows in the right eye point to the congenital retinal macrovessel crossing the foveal avascular zone. (B) Normal fundus of the left eye.

A 19-year-old female patient presented to our department for a routine evaluation. She gave no history of trauma or headache. There was no significant family history. Vision in both eyes was 20/20, N6 unaided. Anterior-segment evaluation and intraocular pressure in both eyes were normal. Fundus evaluation of the right eye revealed a healthy disc with an aberrant vessel originating from the inferotemporal major retinal vein. It crossed over the fovea, giving off multiple branches just above the foveal avascular zone (FAZ). Fundus evaluation of the left eye was within normal limits (Figure 1). Amsler grid test was normal in both eyes.

**Figure 2:**
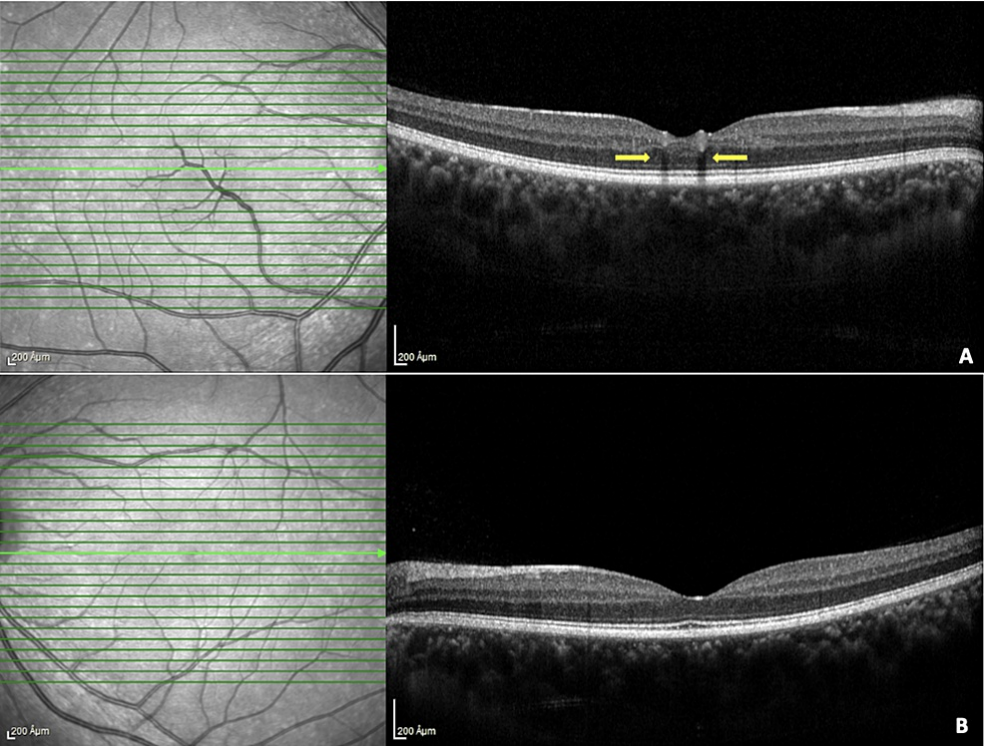
Optical coherence tomography (B scan) of the right (A) and left (B) eyes. Mildly altered foveal contour in the right (A) eye with yellow arrows indicating the shadowing effect of the macrovessel. The normal foveal contour in the left (B) eye.

**Figure 3:**
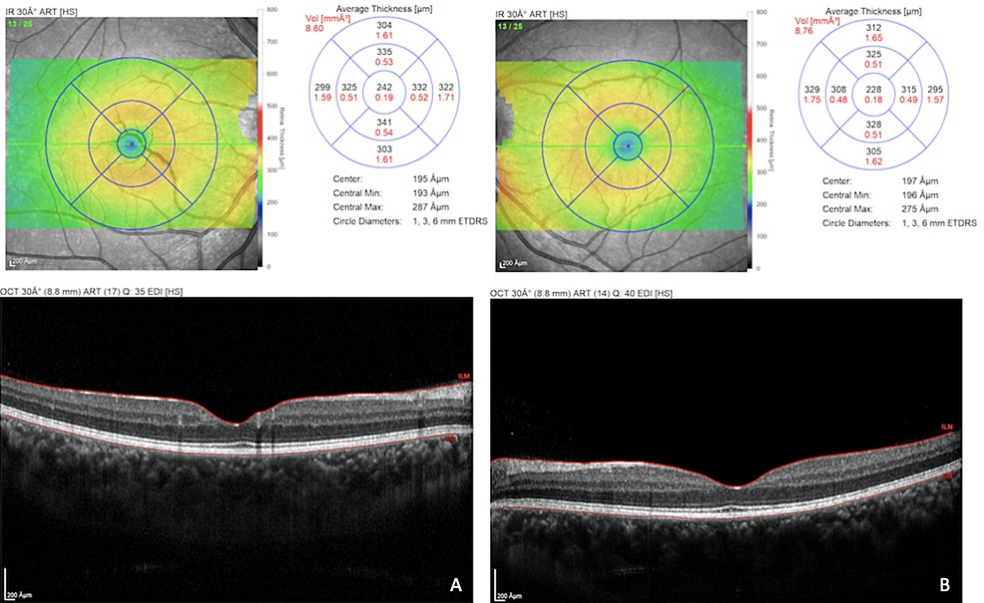
Macular thickness on spectral-domain optical coherence tomography of the right (A) and left (B) eyes. The spectral-domain optical coherence tomography image shows mild parafoveal thickening in the right (A) eye compared to the left (B) eye.

The patient was imaged using Heidelberg Spectralis HRA+ OCT (Heidelberg Engineering Germany). Spectral-domain optical coherence tomography (SD-OCT) of the right eye revealed an altered foveal contour with a highly reflective backscattering vessel across the fovea with shadowing. It was reaching up to the inner nuclear layer. The posterior hyaloid layer was not visualized separately. The foveal contour in the left eye was normal (Figure 2). There was no macular edema; however, mild parafoveal retinal thickening was noted in the right eye compared to the left eye (Figure 3). Central macular thickness was 242 µm in the right eye and 233 µm in the left eye. Due to their low scattering characteristics and the shadowing effect of the vessel wall, the retinal components between the retinal pigment epithelium and the vessel wall were not visible.

**Figure 4:**
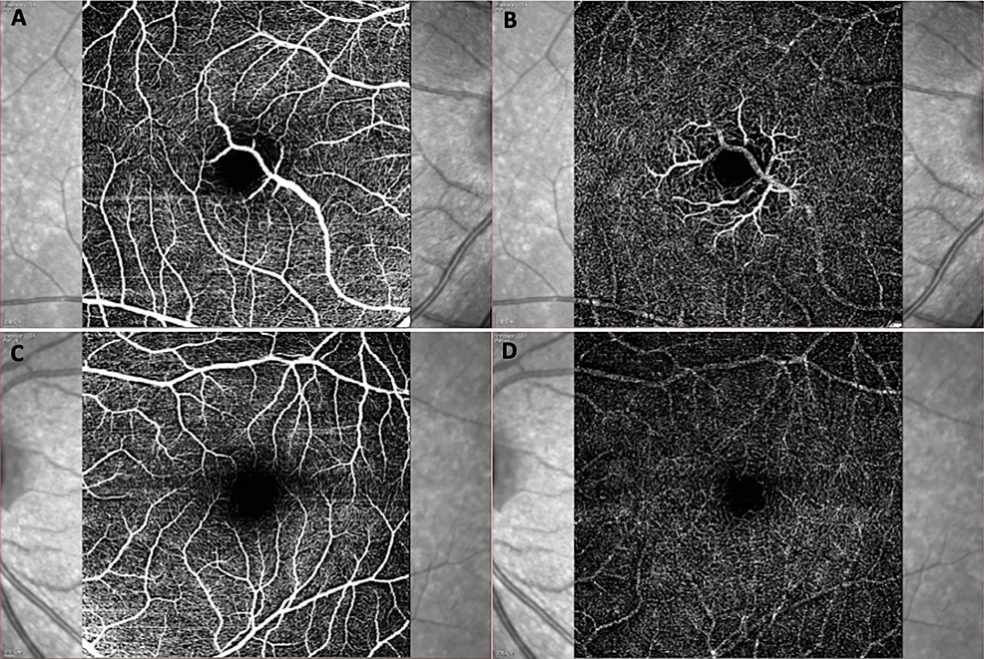
Optical coherence tomography angiography images of the right eye (A and B) (top row) and left eye (C and D) (bottom row). The images indicate the superficial vascular complex (internal limiting membrane to inner plexiform layer) of the right eye (A) and the left eye (C) and the deep vascular complex (DVC) (inner plexiform layer to outer plexiform layer) of the right eye (B) and the left eye (D). (A) demonstrates how the macrovessel branches at the fovea after originating from the inferior temporal vein. The foveal avascular zone is divided diagonally and is distorted as a result. At the level of the DVC, the terminal branches of the aberrant artery are visible.

OCTA (Heidelberg Spectralis; Heidelberg Engineering, Germany) of the right eye revealed the atypical vein originating from the inferotemporal vein and dividing into multiple branches draining from both the deep vascular plexus to the superficial vascular plexus (Figure 4). The maximum diameter of the CRM was 134 µm. There was a mild distortion of the FAZ in the right eye. The maximum diameter of the FAZ measured in the right eye was 902 µm and that in the left eye was 888 µm.

The patient refused to consent to fundus fluorescein angiography (FFA) and magnetic resonance imaging (MRI) of the brain.

## Discussion

Mauthner first described CRM in 1869. In 1969, Ashton explained the formation of CRMs [2,3]. These vessels have mesenchymal origins and appear between 15 and 16 weeks of gestation when arteries and veins begin to differentiate. Normally, high levels of oxygen in the foveal region contribute to the obliteration of the vessels. However, in the case of hypoxia, the proliferation of the vessels may reach the foveola [2]. Brown et al. in 1982 formally described the vessel as a “congenital retinal macrovessel.” This vessel is usually unilateral, abnormally large, supplying or draining the macular region, both inferior and superior to the horizontal raphe [4,5].

CRMs are mostly unilateral, single veins [2,3]. Blood from the superior and inferior retina may be supplied or drained by such aberrant vessels. CRMs are rare and tend to remain stable with visual acuity preserved in most cases [5]. According to the literature, the prevalence of this condition is 1/200,000 [3]. The majority of CRM cases that have been reported in the literature were associated with normal visual acuity, which tends to remain stable for long durations of follow-up [1,3-5]. Brown et al. first reported seven eyes with CRM having normal vision [4]. De Crecchio et al. followed a case with impaired but stable visual acuity over 14 years [6].

Impaired visual acuity has been reported to have been caused by the macrovessel crossing the foveola, foveolar cyst, foveal ectopia, pigmentary changes at the fovea, macular hemorrhage, exudates, or serous macular detachment [4,7,8]. Impaired vision can also be caused by the thickening of the macula and distortion of foveal architecture, as seen on SD-OCT [2] and relative angioscotoma caused by the CRM [9]. Few authors have described central serous retinopathy, retinal cavernous hemangiomas, and retinal venous malformations [7,8,10,11]. OCT in our case revealed an altered foveal contour due to the CRM, shadowing behind the vessel, demonstrating that the highly scattering nature of the CRM prevented the OCT beam from penetrating the deeper layers. This may explain the relative angioscotomas experienced by patients, although not in our case.

Pichi et al. conducted a cross-sectional, multicenter, retrospective study from seven different retina clinics worldwide over a 10-year period. They described 49 patients in their study, of whom 24% had associated vascular malformations of the brain on MRI [10]. They emphasized the importance of systemic workup (including MRI) in patients with CRM. Park et al. described a pediatric patient with CRM with associated asymptomatic cerebrovascular abnormalities [12]. These studies have advocated careful neuroimaging in patients with CRM to reveal other cerebrovascular malformations. Our patient refused to consent to an MRI.

OCTA is a newer, faster, noninvasive modality of evaluation. There is no need for dilatation and fluorescein dye, thus avoiding drug-related contraindications. It also allows the visualization of the superficial and deeper layers separately [13]. The FAZ can be visualized clearly. In our case, the CRM drained from the superficial venous plexus and the deep venous plexus and spanned from the internal limiting membrane to the inner nuclear layer. The presence of CRM caused the distortion of the FAZ, though the FAZ diameter was almost equal to the fellow eye. Previous reports of CRM have documented FAZ that were small or with interocular asymmetry [1,14].

Given the potential impact of CRM on vision, further research is warranted to unravel its molecular and genetic roots, refine diagnostic strategies, and optimize treatment regimens.

## Conclusions

We present a case of CRM in an asymptomatic patient. SD-OCT and OCTA are of immense value in visualizing the architecture of the retinal layers and retinal vasculature and in picking up any vascular abnormalities. These investigations will also be of great value in following up with patients.
